# Perception of risk of HIV and sexual risk behaviors among University students: implication for planning interventions

**DOI:** 10.1186/1756-0500-7-162

**Published:** 2014-03-19

**Authors:** Yitayal Shiferaw, Abebe Alemu, Abate Assefa, Berihun Tesfaye, Etsegenet Gibermedhin, Misiker Amare

**Affiliations:** 1 Department of Medical Microbiology, College of medicine and health sciences, School of biomedical and laboratory sciences, University of Gondar, PO Box ET196, Gondar, Ethiopia; 2 Department of Medical Laboratory science, College of medicine and health sciences, School of biomedical and laboratory sciences, University of Gondar, Gondar, Ethiopia

**Keywords:** HIV risk perception, Risky sexual behavior, Students, Ethiopia

## Abstract

**Background:**

The university environment offers great opportunity for HIV high-risk behaviors, including unsafe sex and multiple partnerships. Despite recently gained decline of the overall incidence of HIV infection, still significant proportion of youth population are at high risk of HIV infection. The aims of this study were to assess the perception of HIV risk and factors associated with risk perception among students at University of Gondar, Northwest Ethiopia.

**Methods:**

A cross sectional study was conducted between February and April, 2012 among health science students. A total of 384 students were involved in the study using stratified sampling technique. Chi-square test and logistic regression analysis were employed. P-value < 0.05 was considered statistically significant for all cases.

**Results:**

Of the total 384 participated students, 200(52.1%) were females. Out of the total study respondents, 202(52.6%) were sexually experienced. One hundred and nine (59.2%) out of 184 males and 93(46.5%) out of 200 females had had sexual experience. About 23(57.5%) of those age below 20 years, 70(52.2%) of 20-24 years old, and 13(61.9%) of those ages of 25 years or older were perceived themselves as if they have no chance of acquiring HIV infection. Students initiated sexual intercourse at early age (≤8 years) were significantly associated with having multiple partnerships (crude OR =3.6, p = 0.002 for male and crude OR = 1.7, p = 0.04 for female). Statistically significant difference was observed in the distribution of condom use during sexual intercourse among various age groups (p-value = 0.001). Sexual initiation at younger age, having multiple partnerships, inconsistent condom use and alcohol and/or drug abuse were significantly perceived as predictor for an increased risks for HIV infection.

**Conclusion:**

Students were engaged in various HIV risk behaviors. Early sexual initiation and alcohol and/or drug abuse were important factors for having multiple partnerships. Poor agreement between having HIV risk behaviors and perception of HIV risk were observed. Attention has to be given on the role of alcohol and/or drug abuse in the participation of HIV risk behaviors in the design and implementation of HIV prevention for university students.

## Background

Since 1981 where the first case of human immunodeficiency virus (HIV) was recognized in the United States, HIV has spread rapidly throughout the world. According to report at the end of 2005, the number of people living with HIV was 38.6 million. An estimated 4.1 million were newly infected with HIV and 2.8 million lost their lives due to acquired immunodeficiency syndrome (AIDS) [1].

The first case of HIV in Ethiopia was reported in 1984. Since then HIV/AIDS become a major public health concern in the country leading the Government of Ethiopia to declare a public health emergency in 2002. In 2007, the estimated adult HIV/AIDS prevalence in Ethiopia was 2.1%. National projections estimated approximately 1.1 million Ethiopians were living with HIV and prevalence was increase slightly to 2.3% by 2009 [2]. At the regional level, in Amhara region, the prevalence rate of HIV (4.5%) is highest next to two administrative cities, Addis Ababa (11.7%) and Dire Dawa (6.8%), and the dominantly urban region (Harari) (5.2%) [3]. Although the epidemic is currently stable, HIV/AIDS remains a major development challenge for Ethiopians. Poverty, food shortages and other socio-economic factors are amplified the impact of the epidemic [2].

Over half of all new infections worldwide were among young people between the ages of 15 and 24 years. Every day, 6,000 young people become infected with HIV more than five every minute [4]. In the United States, half of all new infections are estimated to be among people under age 25 years and the majority of young people are infected sexually [5]. Demographic health surveys of many countries were revealed adolescents are experienced puberty nowadays at younger age than the previous generation. As result, they are involved in early initiation of sexual intercourse; most of it is being unsafe, unplanned and exposing them to unwanted pregnancy, abortion and sexual transmitted disease [6].

Globally, university students are in the age range with the highest rates of new HIV infections [7]. The university environment with its attendant relative lack of parental supervision offers great opportunity for young people, who are bridging from adolescence to adulthood, to test the limits of their new found freedom through sexual experimentation [7,8]. Such experimentation frequently involves engagement in risky sexual activities such as multiple partnerships, inconsistent use of condoms, and having sex under the influence of alcohol or drugs [9]. Literature on health related behavior emphasizes the perception of being at risk of infection as one of the necessary conditions for behavioral change [10]. Moreover, the degree of the perceived risk seems to affect individual actual control in adopting preventive measures. Individual risk perception as well as individual knowledge, is likely to be subjected to socio-environmental influences, as long as social interaction allows information exchange, facilitates common evaluation and definition of the meaning and of its validity [11].

Interventions developed for general population may not be appropriate for university students. In order to develop the body of knowledge necessary needed to develop interventions targeted to different category of people [7], it is an immense important to study the overall HIV risk behaviors and perception of risk among university students. This approach is critical because of the plausible differences in risky behaviors between on-campus and off-campus students [12]. University students are at risk because they tend to be sexually adventurous, often with multiple partners and do not consistently use condoms [7,13]. Risky sexual behavior includes early age at first sexual intercourse, multiple sexual partners, unprotected sexual intercourse, and untreated sexually transmitted diseases [14]. In both low and high endemic settings, reducing the vulnerability of young people to HIV infection is the principal defense against the epidemics of the future [11]. Although various surveys have been done to study the sexual behavior of youth in Ethiopia, very few studies have assessed youth HIV risk sexual behaviors and perception of HIV risk. So far no study has been carried out on perception of HIV risk and sexual risk behaviors among university students in the study area. Fully understanding these issues will help to provide a foundation for future inquires into the behavioral aspects that contribute to HIV transmission. Hence, assessing the perception of HIV risk and sexual risk behaviors among students will provide information for policy makers to design and implement appropriate programs on prevention of HIV transmission among university students.

## Methods

### Study area and design

A cross sectional study was conducted from February to April, 2012 among health Science undergraduate students at University of Gondar (UoG). The University is found in Gondar town. Gondar town is located in the North Gondar Zone of the Amhara Region, Northwest Ethiopia, and 748 km far from Addis Ababa, the capital city of Ethiopia. The university has different colleges and schools having many departments.

### Study population and sampling procedure

There were a total of 2,500 health science undergraduate students at UoG during the study period. The sample size was determined using single population proportion statistical formula taking 50% HIV high-risk behaviors because of absence of similar study in the study area. Considering 5% of absolute precision with 95% confidence interval, a minimal sample size of 384 was attained. Assuming homogeneity in academic status of the students in the same year of study, stratified proportionate sampling was used to select study participants. Students’ list was obtained from student registration books and based on their year of study stratified into six categories (year І to year VІ undergraduate students). From each category proportional numbers of students were selected using systematic random sampling to obtain the total samples for the study.

### Data collection

Face-to-face survey using self-administered questionnaires were conducted to obtain the following information from the study participants: socio-demographic characteristics, sexual behaviors, comprehensive knowledge on HIV/AIDS and risk perception of HIV infection. The questionnaires were adapted from survey instruments that have been tested and used to conduct college based HIV/AIDS knowledge, attitudes, beliefs and behavior surveys [12,15-17]. In this manuscript, only the data relating to sexual risk behaviors and perception of HIV risk are presented. In order to see the reliability of the adopted questionnaires, the English version was translated into simple Amharic (local language) and back translated into English. And in the process there was no significant difference between the two versions and the local language was used to collect the required information. Pre- test of questionnaires were conducted on twenty students and the results were used to improve the phrasing of questions in the questionnaires.

### Measures

We defined “sexually experienced” as having previously or currently engage in any form of sexual practices. The two HIV-risk sexual behaviors of multiple sexual partners and inconsistent or non-use of condoms were taken from CDC’s list of risk factors for HIV transmission [18]. Participants were categorized as having multiple partners if they had more the one partners within the past 6 months. Consistency of condom use was assessed by the question: “During the past 6 months, how often did you or your partner use a condom? Always was scored “0”, any other answer was scored “1” (risky sex). The consistency of condom use among sexually experienced students who did not have sex within the last 6 months prior to the study was assessed by the question: “The last time you had sexual intercourse, did you or your partner use a condom?” Using condom always is coded as “consistent condom use” and other levels of condom use (rarely, sometimes, most of the time) or non-use as “inconsistent condom use”. Self-perception of HIV risk was assessed by the question “what are the chances that you might catch HIV? Would you say there is no chance, a moderate chance or a good chance”? For the analysis, ‘moderate or good chance’ was scored as 1, and no chance or don’t know were scored as 0. In our analysis, low perception is defined as ‘no chance’ and high perception as ‘moderate/good chance’.

### Data analysis

Data coding, cleaning and verification were performed to ensure quality of data before analysis. Data were entered and analyzed using SPSS software version 16. Descriptive analysis was conducted on the demographic variables such as age, ethnicity, religion, sexual experience, year of study and HIV risk perception. HIV risk behaviors and perception of the risk of HIV infection were compared between the different age groups using Chi-square (*χ*2) tests. Bivariate and multivariate logistic regression analyses were performed to determine the degree of association of HIV risk perception with the independent variables of age, ethnicity, religion, sexual experience and year of study. To control simultaneously for the possible confounding effects of the different variables, possible risks were estimated by multivariate analysis. Odds ratio (OR) and 95% confidence intervals (CIs) were calculated. P-values < 0.05 were considered statistically significant in all cases.

### Ethical consideration

Ethical clearance was obtained from University of Gondar collage of Medicine and Health Science School of Biomedical and laboratory Science Ethical Review Committee. Prior to data collection, the purpose of the study was explained for participants and the confidentiality of the results was assured. All respondents were signed the informed consent form before participation.

## Results

### Socio-demographic characteristics

Of the 384 study participants, 200(52.1%) were females. The age of the study participants were ranged from 19 to 26 years with a mean of 21. 5 ± 5 SD years. The majority of the students were in the age group of 20-24 years 235(61.2%). In terms of ethnicity, most students were Amhara 165(43%) and majority of them were first year students 98(25.6%). All the students were not married (Table 1).

**Table 1:** Socio-demographic characteristics of the study participants

**Categories**		**Number**	**Percentage (%)**
Age in years	<20	76	19.8
	20-24	235	61.2
	≥25	73	19
Gender	Male	184	47.9
	Female	200	52.1
Ethnicity	Amhara	165	43
	Oromo	63	16.4
	Tigray	48	12.6
	Others	108	28
Religion	Orthodox	276	71.9
	Muslim	56	14.5
	Others	52	13.6
Sexual experience	Yes	202	52.6
	No	182	47.4
Year of study	I	98	25.6
	II	88	23
	III	79	20.5
	IV	62	16.1
	V	40	10.4
	VI	17	4.4

### Sexual characteristics

Of the total respondents, 202(52.6%) were already have a previous history of sexual experience and 5(2.5%) of them had their sexual debut at the age of 16 years or younger, 70(34.6%) between 17 to18 years and 127(62.9%) at age 19 years or older. Moreover, of the sexually experienced participants, 8(20%) of those who are under the age of 20 years, 51(36.2%) between 20 and 24 years, and 6(28.6%) of those 25 years and above were reported having sexual intercourse two or more times within the last 6 months. In this study, Pearson chi-square analyses were showed statistically significant differences in the distribution of condom use during sexual intercourse among the various age groups (p = 0.001) (Table 2). The perception of the risk of HIV infection was generally low among students. About 23(57.5%) of those age below 20 years, 70(52.2%) of 20-24 years old, and 13(61.9%) of those ages of 25 years or older were perceived themselves as if they have no chance of acquiring HIV infection (Table 2).

**Table 2:** Chi-square analysis of sexual behaviors and perception of HIV risk of 202 sexually experienced students by age

**Categories**		**Age in years**			**p-value**
		**≤19 (n = 40)**	**20-24 (n = 141)**	**≥25 (n = 21)**	
Frequency of sexual intercourse in the last 6 months	0	21	50	8	0.21
	Once	11	40	7	
	≥2 times	8	51	6	
Number of sexual partners for female	1	15	51	8	0.255
	≥2	3	14	3	
Condom use in last sexual intercourse	Yes	25	55	5	0.01
	No	15	86	16	
Condom use during sexual intercourse in the last 6 months	Never/rarely	10	75	16	0.001
	Sometimes	7	33	2	
	Always	23	33	3	
ADULSI	Yes	14	90	13	0.21
	No	26	51	8	
Perceived HIV risk	No	21	68	12	0.96
	Moderate	13	45	6	
	Good	4	19	2	
	Don’t know	2	2	1	

AULSI: Alcohol and/or drug use in the last sexual intercourse.

Of the 202 sexually active respondents, 109(54%) were females while the remaining 93(46%) were males. The majority of the sexually active female respondents 64(58.7%) had multiple sexual partners within the last 6 months before the commencement of the study. However, only 18(19.4%) males had multiple sexual partners. Females who used alcohol along with illegal drugs and low educational levels (1^st^ -3^rd^ year students) were significantly associated with having multiple sexual partners (COR 2.2, 95% CI 1.49 -3.31, p = 0.001 and COR 1.8, 95% CI 1.24 to 2.6, p = 0.001) respectively. Moreover, students initiated sexual intercourse at early age (≤18 years) were significantly associated with having more than one partner (COR 3.6, 95% CI 1.50 -8.79, p = 0.002 for male) and (COR 1.7, 95% CI 1.02 - 1.52, p = 0.04 for female) compared to those who are initiated sexual activity age above 18 years (Table 3).

**Table 3:** Bivariate analysis of risk factors for having multiple sexual partners in the previous 6 months

**Variables**		**Female with multiple sexual partner (n = 64)**			**Male with multiple sexual partner (n = 18)**		
		**n (%)**	**COR (95% CI)**	**p-value**	**n (%)**	**COR (95% CI)**	**p-value**
Age in year	<20	14(63.6)	1	0.11	3(16.7)	1	0.579
	20-24	47(1.8)	0.93(0.346,2.48)		14(21.5)	1.62(0.41,6.33)	
	≥25	3(27.3)	0.21(0.04,1.05)		1(10)	0.63(0.06,6.96)	
Ethnicity	Amhara	28(59.6)	1	0.898	10(25)	1	0.374
	Oromo	11(61.1)	1.07(0.35,1.05)		1(6.7)	0.21(0.03,1.84)	
	Tigray	9(64.3)	1.22(0.35,4.22)		1(8.3)	0.27(0.03,2.39)	
	Others	16(53.3)	0.78(0.31,1.95)		6(23.1)	0.9(0.28,2.87)	
Year of study	1-3	73.3	1		10(15.6)	1	
	4-6	40.8	1.79(1.24,2.60)	0.001	8(27.6)	1.17(0.91,1.49)	0.176
ADULSI	Yes	58(68.3)	2.22(1.49,3.31)	0.001	5(14.3)	0.58(0.186,1.77)	0.336
	No	6(29.6)	1		13(22.4)	1	
Age at sexual debut (in year)	≤18	35(77.8)	1.7(1.02,1.52)	0.04	12(40)	3.64(1.50,8.79)	0.002
	≥19	29(45.3)	1		6(9.5)	1	
UIDA in the last 6 months	No	9(34.6)	1		8(14.3)	1	
	≥1 times	55(66.3)	1.94(1.29,29.24)	0.04	10(27)	1.16(0.94,1.47)	0.128

AULSI: Alcohol and/or drug use in the last sexual intercourse, UIDA: Use of illegal drugs and alcohol, n: the number of respondent reporting multiple partnerships, COR: Crude odds ratio.

### Condom use

Table 4 showed the relationship between dependent variables (inconsistent condom use in the last 6 months) and selected explanatory variables. For this analysis, we separated the results by gender considering differences in engagement of HIV risk behaviors between males and females. Of the total sexually experienced students, 60(55%) males and 93(89.2%) females were reported inconsistence condom use in the last 6 months. The distribution of condom use in the last sexual intercourse varies between age groups as 25(62%) of those age ≤19 years, 55(39%) of those age 20-24, and 5(23.8%) of those age 25 years and older were used it. This study reviled inconsistent condom use in the last 6 months increased as the age of the participants were increased. Logistic regression analysis indicated that sexually experienced male students of ≥ 25 years old and those 20-24 years were significantly associated with inconsistent use of condom (COR 1.8, 95% CI 2.07-16.28, p = 0.006; COR 2.3, 95% CI 1.30-15.53, p = 0.006 respectively) compared to those age below 20 years old. Similarly, among females, those age 25 years and older (COR 1.8, 95% CI 2.07-16.28, p = 0.002) and age between 20-24 years (COR 2.02, CI 95% 1.30-15.53, p = 0.002) were more likely use condom inconsistently compared to those age below 20 years old. Moreover, there was statistically significant association between drinking alcohol along with illegal drugs and inconsistence condom use among female students (COR 2.2, p = 0.00) (Table 4).

**Table 4:** Bivariate and multivariate analysis of risk factors for unprotected sexual intercourse among sexually experienced students by gender

**Variables**		Inconsistence condom use in the last 6 months*				
		**Male (n = 109)**		**Female (n = 93)**		**AOR (95% CI)**
		**(%)**^**a**^	**COR (95% CI)**	**(%)**^**a**^	**COR (95% CI)**	
Age in year	<20	38.9	1	45.5	1	1
	20-24	69.2	1.8(1.59,13.5)**	83.0	1.8(2.07,16.28)**	1.8(2.08,16.28)**
	≥25	90.0	2.3(1.74,14.3)**	90.9	2.02(1.30,15.53)**	2.02(1.30,15.53)**
Ethnicity	Amhara	72.5	1	80.9	1	1
	Tigray	60.0	0.3(0.09,1.13)	61.1	0.24(0.073,0.77)	0.24(0.073,0.77)
	Oromo	58.3	1.3(0.25,4.99)	64.3	0.80(0.2,3.77)	0.81(0.2,3.77)
	Others	57.9	0.5(0.18,1.4)	83.3	1.18(0.35,3.95)	1.18 (0.35,3.95)
Year of study	1-3	65.6	1	80.0	1	1
	4-6	62.1	0.85(0.34,2.13)	71.4	0.63(0.26,1.52)	0.625(0.258,1.52)
ADULSI	Yes	62.9	0.89(0.37,2.13)	87.8	2.2(3.80,14.85)	2.5(4.11,15.70)**
	No	65.5	1	40.7	1	1
Age at sexual debut	≤18	73.3	1.2(1.06,6.30)	66.7	0.42(0.17,1.02)	0.42(0.17,1.02)
	≥19	60.3	1	82.8	1	1
UIDA in the last 6 months	No	62.5	1	50.0	1	1
	≥1 times	67.6	1.25(0.52,3.00)	84.3	1.21(0.39,3.76)	1.21(0.39,3.76)

ADULSI**:** Alcohol and/or drug use in the last sexual intercourse, ^a^percent of inconsistent condom use in the last 6 months among sexually experienced students, *Adjusted for all variables listed in the tables, **p ≤ 0.05, COR: Crude odds ratio, AOR: Adjusted odds ratio.

### HIV risk perception

In this study, the perception of the risk of HIV infection was generally low among students. Only 106(52.5%) of those respondents who are inconsistently used condoms in the previous 6 months were perceived themselves having moderate to good chance of acquiring HIV infection. Among sexually experienced respondents, 49(54.9%) were perceived multiple partnerships as a risk of HIV infection. Moreover, as shown in Table 5, sexual initiation at younger age, having multiple partnerships, inconsistent condom use and alcohol and/or drug abuse were significantly perceived as predictor for an increased risks for HIV infection (Table 5).

**Table 5:** Bivariate and multivariate analysis of factors associated with reporting moderate to good perception of HIV risk among sexually experienced respondents

**Variables**		**Moderate/good perception of risks for HIV infection (n = 89)**		
		**(%)**^c^	**Odds ratio with 95% CI**	
			**Crude OR (95% CI)**	**Adjusted OR (95% CI)**
Age at sexual debut (in year)	≤18	36.0	1	1
	≥19	52.6	2.69(1.47,1.94)**	2.69 (1.47,1.94)**
Condom use in last 6 months	Consistence	23.7	1	
	Inconsistence	52.5	3.54(1.78,7.02)**	3.75(1.5,9.02)**
UIDA in the last 6 months	Yes	54.2	2.86(1.57,5.18)**	2.3(1.31,4.33)*
	No	29.3	1	
Multiple partnership	Yes	54.9	2.1(1.19,3.72)**	1.8 (1.13,3.51)*
	No	36.7	1	

UIDA: Alcohol and/or drug use in the last sexual intercourse, ^c^percent of sexually experienced students who perceived good/moderate HIV risk, *p < 0.05, **p < 0.01.

## Discussion

### Sexual behavior and perceptions of HIV risk

This study tried to give important information regarding the sexual behavior of university students, their risk perception, and possible protective measures including condom use that should be implement in an effort to control the spread of HIV. In this study, 52.6% of the participating students were admitted previous history of sexual experience which comprises, 50.4% of boys and 54.5% of girls. These figures are almost in line with the results of similar studies in Kenyan [19], Uganda [20], in cities’ of Ethiopia such as Addis Ababa [21], Bahir Dar [22], and Harar [23]. Young people in both developing and developed countries commence sexual activity relatively early ages. In this study, most of the participants were initiated their first sexual intercourse at the age range of 19 years or more. It was also observed, 2.5% of those who already commence sexual intercourses were initiated the first act at the age of 16 years or less. Comparable low rate of sexual initiation at early age was reported in South Africa but similar study in USA high rate of sexual initiation at early age was reported [24].

Having multiple sexual partners is a recognized risk for HIV infection. We observed an overall rate of 40.6% multiple partnerships among sexually experience respondents. Similar comparable findings are reported in different studies [19,24,25]. The majority sexually active respondents and having multiple partnerships were found to be female students. In contrast to these findings, report from Uganda indicated larger proportion of males (62.9%) than females (51.3%) were sexual activity and more males (40.5%) than females (25.0%) had multiple sexual partners [26]. Significant numbers of students who are initiated sexual activities at early age in both sexes were more likely to have multiple sexual partners. Literature support early sexual debut has been associated with HIV-related risk factors like multiple partners, unprotected sex and alcohol use in the context of sexual intercourse [14,27,28]. In contradict to the report from Uganda [26], fewer cases of multiple partnerships were observed among students of older age group in both sexes. This finding was comparable to similar studies [19,25]. These findings may be attributed to the “maturing out” phenomenon. This phenomenon is believed to result when adolescent transit to adulthood and take responsibilities. And these events are associated with change in attitudes toward problematic sexual behavior and HIV risk perceptions. Overall, respondents who are reported alcohol or illegal drug use in the last sex and in the previous 6 months before the study were more likely in all cases to have multiple sexual partners. Once again this suggests that individuals with one HIV risk behavior are more likely to exhibit others.

Another characteristic feature that makes youth sexual activity high risk is their either non-or very minimal use of any protective measures, specifically the use of condom. In keeping with other findings [25,29,30], this study found consistent use of condoms to protect individuals from HIV infection was low among sexually experienced respondents of all ages. More than half (57.9%) of sexually experienced respondents were not utilize condom in the last sex and only 29.2% were claimed consistent condom utilization during subsequent sexual encounters. We also observed an age-related downward trend of consistent condom utilization where as age increased condoms utilization was decreased. Respondents aged 25 years or older significantly more likely than those below 20 years to report that they or their partners were not use condom during last sex and less likely to report they or their partners were always used condom during sexual intercourse in the last 6 months. The same age group was less likely to report having more than one sexual partner. As a result of this contradiction, any protective effect granted by having single partnership is eroded by lack of condom use because their partners may also have other partners.

Female students also significantly more likely than males to report them or their partners were not use condom during last sex and /or within the last 6 months. Low condom use among females is quite troubling because women are more likely than men to be infected with HIV during sexual intercourse. In addition females in our sample were more likely reporting having multiple sexual partners. These make a twofold risk in transmission of HIV infection compared to male. Moreover, as a group, students who use alcohol and/or illegal drugs were also more likely to report having multiple sexual partners and inconsistent condom use. These findings were concordant with previous studies [31,32] that have shown individuals engaging in HIV risk behaviors often engage in other high risk behaviors. These findings will indicate more attention needs to focus on the role of alcohol and drug use in the participation of HIV sexual risk behaviors in the design and implementation of HIV prevention programs for university students.

### Factors associated with risk perception

Perception of risk may be a strong motivating factor for behavioral change, particularly if the individual perceives control over the risk behavior [33]. The tendency to systematically underestimate personal risk termed ‘optimistic bias [34] and treating HIV infection as a distant possibility [35] have been reported among college students. This could be due to adoption of protective behaviors, which is unlikely to occur unless the person is well aware of the risk of HIV infection. In the case of the level of HIV infection associated risk sexual behaviors among our sample students, self-perception of HIV risk was low. About 55.9% of sexually experienced students were not perceived themselves as a potential target for HIV infection. The risk here is with their low perception of potential candidate for HIV infection; students may not take any HIV preventive measures at the time of exposure to risk sexual behaviors. Study showed that people can judge their risk of HIV infection [36]. However, sometimes people who are at risk may not perceive their risk and are less motivated to protect themselves [37]. Even though the number of students who are reported alcohol and/or other drug use; multiple partnerships; and inconsistent condom use were high, most strikingly students who have these risk behaviors were significantly perceived themselves as having a moderate to good risk of being infected with HIV than they do not. These observations implies that students who lead risk free sexual behavior, believe and expect their partner also do the same and gradually become reluctant and less motivated to apply prevention methods all the time in future. Current good students’ sexual behavior cannot be a guaranty for its continuation and it is difficult to be confidence enough and rely on students living in area where high risk sexual behaviors were abundant. Therefore, students’ risk perception for HIV infection could be there whether there is risk sexual behavior or not.

As results of globalization and easy access of technology the rate of communication and information delivery have been improved among the society. Social media like face book which attract attention of youth should be implement to transmit prevention messages periodically. College student television broadcasts, use of group SMS message, students’ newspapers are possible approaches which should be consider while designing HIV prevention interventions for university students. All these communication channels should emphasize importance of condom use, risk behind multiple sexual partners, effects of alcohol and drugs for HIV related risk behaviors and the importance of once HIV risk perception in control of its transmission. It is also advisable providing of free condoms in college or student residence hall.

### Limitation of the study

Sensitive and unethical issues like socio-cultural and partner’s emotional and psychological characteristics of the participants which can influence HIV risk perception and behavior were not collected. These make this study to be deficient in assessing all factors responsible for HIV risk perception and behavior. Moreover, since this is a cross-sectional study, the direction of causal relationships cannot be determined.

## Conclusion

This study suggests that many participants were engaged in risk behaviors that exposed them to HIV infection. The engagement of the students in HIV sexual risk behaviors were varied by age and gender. Early age sexual initiation and alcohol and/or drugs use were important factors for having multiple partnerships. Condom use was generally poor among older students and use of alcohol and/or drug was important predictor for inconsistent condom use. Furthermore, we identified poor agreement between engaging in HIV risk sexual behavior and perception of HIV risk among the students. The findings suggest that college students cannot be considered a homogenous population for which one type of intervention will be effective. As a results intervention programmes for HIV prevention in this University should be prepared in a manner of addressing different issues in various categories of students based on the observed deficiencies. It is better to give more attention on the role of alcohol and drug use for the contributions of HIV sexual risk behaviors in the design and implementation of HIV prevention for university students.

## Consent

Written informed consent was obtained from the study participants for the publication of this report.

## Competing interests

The authors declare that they have no competing interests.

## Authors’ contributions

YS, BT, EG and MA conceived the study, undertook data collection, undertook statistical analysis and drafted the manuscript. AA and AAs initiated the study and made major contributions to the study design and statistical analysis. All authors contributed to the writing of the manuscript and approved the submitted version of the manuscript.
